# Management of Snakebite-Induced Thrombotic Microangiopathy (TMA) With Plasmapheresis

**DOI:** 10.7759/cureus.50104

**Published:** 2023-12-07

**Authors:** Takshak Shankar, Nidhi Kaeley, Mukund Rajta, Ashwani Pundir, Aseem Kaushik

**Affiliations:** 1 Emergency Medicine, All India Institute of Medical Sciences, Rishikesh, Rishikesh, IND

**Keywords:** acute kidney injury, therapeutic plasma exchange (tpe), hemodialysis, thrombotic microangiopathy (tma), snake-bite

## Abstract

Snakebites affect a lot of people in India. Of these, the hemotoxic snakebites may induce a consumptive coagulopathy, which has been termed now as “Venom-Induced Consumptive Coagulopathy” (VICC). Some patients with VICC develop Thrombotic Microangiopathy (TMA). The primary end-organ damage in TMA is renal, for which hemodialysis is the mainstay of treatment. Recently there has been some focus on plasma exchange as an adjunctive treatment for TMA. Here we present a case of a young male who developed snakebite-induced TMA and who was successfully managed with plasma exchange.

## Introduction

Snakebite was designated as a Neglected Tropical Disease (NTD) by the World Health Organisation (WHO) in 2017 [1]. The National Health Profile of India reported 164,031 cases of snakebites and 885 deaths resulting from snakebites in the year 2018 [2]. The major contributor to snakebite mortality is haemorrhage [3]. The typical procoagulant coagulopathy induced by snakebite is called Venom-Induced Consumptive Coagulopathy (VICC). Certain patients with VICC develop Thrombotic Microangiopathy (TMA), which is characterized by Microangiopathic Hemolytic Anemia (MAHA) and Thrombocytopenia. MAHA is characterized by the presence of schistocytes on the peripheral blood smear [4-6]. Of late, there is a considerable focus on plasma exchange therapy as an adjunctive treatment in snakebite-induced TMA.

## Case presentation

A 26-year-old male with no known comorbidities and addiction history presented to the emergency department (ED) with an alleged history of snake bite on his right great toe when he was working in the fields. He developed swelling of his feet within a few minutes of the bite associated with pain. There was no blistering at the bite, and the swelling was to the ankle at presentation. He had an episode of hematuria around two hours after the bite, following which he had no urine output. There was no history of vomiting, abdominal pain, headache, blurring of vision, drooping of eyelids, weakness, difficulty in swallowing or bleeding from any other site.

The patient was conscious and oriented to time, place, and person on examination. His single breath count was more than 30. Initial vitals were a pulse rate of 100/min, blood pressure of 140/90 mmHg and respiratory rate of 20 breaths per minute. Initial oxygen saturation was 88% at room air which improved to 96% with 4 litre oxygen via nasal prongs. The neurological examination revealed a power of 5/5 in both upper and lower limbs with normal tendon reflexes and intact sensation. Respiratory examination revealed bilateral basal crepitations on auscultation. Other system examinations were within normal limits. A bedside 20-minute Whole Blood Clotting Test was positive (uncoagulated). Laboratory examination revealed acute kidney injury, as shown in Table 1.

**Table 1:** Laboratory investigation results TLC: Total Leucocyte Count; SGOT : Serum Glutamate Oxaloacetate Transaminase; SGPT : Serum Glutamate Pyruvate Transaminase; PT : Prothrombin Time; INR : International Normalised Ratio; CPK-NAC : Creatine Phosphokinase; LDH : Lactate Dehydrogenase

Laboratory Parameters	Reference Range	Day 1	Day 3	Day 5
Hemoglobin (grams per decilitre)	13-17 g/dl	13.5	7.9	8
TLC (cells per cubic millimetre)	4-11	29	10.6	9.56
Neutrophils (%)	40-70%	87.2%	79.4%	72.6%
Platelet count (thousands per cubic millimetre)	150-400	6	57	64
Serum potassium (millimoles per litre)	3.5-5.1	5.5	3.9	4.01
Serum sodium (millimoles per litre)	136-146	130	136	141
Urea (milligrams per decilitre)	17-43	161	138	121
Creatinine (milligrams per decilitre)	0.72-1.18	5.44	4.69	3.60
Total bilirubin (milligrams per decilitre)	0.3-1.2	1.4	1.2	1.1
Direct bilirubin (milligrams per decilitre)	0-0.2	0.27	0.21	0.12
SGOT (Units per Litre)	0-50	291	150	90
SGPT (Units per Litre)	0-50	79	60	45
PT (seconds)	13.2	20.2	14.3	13.6
INR	1.23	1.93	1.34	1.27
Serum albumin (grams per decilitre)	3.5-5.2	3.3	3.5	3.5
D-Dimer (milligrams per decilitre)	0-0.5	>5.5 mg/dl		1.31
CPK-NAC (Units per Litre)	0-171	6910		264
Fibrinogen (milligrams per decilitre)	180-350	78.4		248
LDH (Units per Litre)	0-248	768	532	310
Peripheral Blood Smear		Schistocytes (55%) present		

The patient was administered 30 vials of polyvalent anti-snake venom as per the National Guidelines for the treatment of snake bites. He was also initiated on intravenous antibiotics, limb elevation, and other supportive measures. He was also transfused 1 unit of packed red blood cells. The patient was started on hemodialysis for oliguric acute kidney injury and fluid overload leading to non-cardiogenic pulmonary edema. Owing to the additional presence of schistocytes on peripheral blood smear and thrombocytopenia, a diagnosis of snakebite-induced TMA was also made. The patient was initiated on plasma exchange therapy for the same on day 3. He underwent three sessions of plasma exchange on alternate days with fresh frozen plasma as the replacement fluid. He also received three sessions of hemodialysis. His local swelling subsided on day 7. His blood parameters and urine output improved and he was discharged on Nephrology follow-up on day 11, with serum creatinine a value of 2.1 mg/dL (reference range 0.72-1.18 mg/dL). His renal parameters normalised completely within a month and he was dialysis-free.

## Discussion

There are over 300 species of snakes in India, out of which only 60 are venomous or mildly venomous. However, the majority of the bites in India result from four snake species, namely Daboia russelii (Russell's viper), Naja naja (common Indian Cobra), Bungarus caeruleus (common krait) and Echis carinatus (saw-scaled viper) [7]. Viperine bites can result in local symptoms in the form of local necrosis, ecchymosis, blistering, painful progressive swelling and compartment syndrome and systemic symptoms in the form of bleeding, shock and Disseminated Intravascular Coagulation (DIC) [8]. The consumption coagulopathy resulting from snakebite is now referred to as VICC and it results from activation of the clotting pathways by the venom procoagulant toxins [4].

Some snake envenomations with VICC develop thrombotic microangiopathy (TMA), which is characterized by small vessel micro-thrombosis and endothelial damage. Microangiopathic hemolytic anemia develops, which is characterized by schistocytes in the blood. The diagnosis of TMA is established by the presence of thrombocytopenia and MAHA primarily and less commonly by tissue biopsy. Vaso-occlusive organ damage is the primary risk in TMA, and in TMA following snakebite, the primary end-organ damage is renal, for which hemodialysis remains the mainstay of therapy [4-6].

Plasma exchange is a non-specific extracorporeal technique that removes plasma components such as metabolites, inflammatory mediators, and toxins. The clinical applications of plasmapheresis are increasing, especially in toxicology [9]. Snakebite-induced TMA has been compared to other TMA conditions such as Thrombotic Thrombocytopenic Purpura (TTP) and Hemolytic Uremic Syndrome (HUS), and due to the predominant renal involvement, it is said to resemble HUS. However, there is a tendency for renal recovery in snakebite-induced TMA, which differentiates it from complement-mediated TMA. While the mainstay of treatment of TTP remains plasmapheresis, plasmapheresis is usually unsuccessful in HUS [10-12]. Nevertheless, plasmapheresis has been reported to be effective in snake bite TMA [13,14]. The current American Society for Apheresis guidelines (ASFA) consider plasmapheresis to be a category III, grade 2C recommendation therapy for the treatment of snake bite envenomation [15].

Our patient was a young male who developed hemotoxic and cytotoxic symptoms after snakebite envenomation. The snake, unfortunately, could not be identified due to the lack of a clear description and photograph. The patient developed an acute kidney injury for which he was started on hemodialysis. The acute kidney injury could be attributed to rhabdomyolysis, but the peripheral blood smear was suggestive of schistocytes and the patient also developed thrombocytopenia, so a diagnosis of snakebite-induced TMA was also made. For this, the patient was started on plasma exchange therapy. A total of three cycles of plasma exchange therapy were received, and an improvement in blood parameters and urine output was seen.

## Conclusions

Snakebite-induced TMA is an incompletely understood entity. However, its timely diagnosis is crucial as it facilitates early treatment. The primary end-organ damage due to TMA in snake bites is renal, for which the mainstay of treatment is hemodialysis. Owing to primary renal end-organ damage, the TMA is said to resemble HUS; however, there are certain differences between snake bite-induced TMA and HUS. This case highlights a beneficial effect of plasma exchange therapy in snakebite-induced TMA, and thus, plasma exchange can be considered as an adjunctive treatment for this entity.
